# Risk of breast cancer after false‐positive results in mammographic screening

**DOI:** 10.1002/cam4.646

**Published:** 2016-02-25

**Authors:** Marta Román, Xavier Castells, Solveig Hofvind, My von Euler‐Chelpin

**Affiliations:** ^1^ Department of screeningCancer Registry of NorwayOsloNorway; ^2^ National Advisory Unit for Women's HealthOslo University HospitalOsloNorway; ^3^ Department of Epidemiology and EvaluationIMIM (Hospital del Mar Medical Research Institute)BarcelonaSpain; ^4^ Network on Health Services in Chronic Diseases (REDISSEC)BarcelonaSpain; ^5^ Oslo and Akershus University College of Applied SciencesFaculty of Health ScienceOsloNorway; ^6^ Department of Public HealthUniversity of CopenhagenCopenhagenDenmark

**Keywords:** Breast neoplasms, false‐positive reactions, mammography, mass screening, risk factors

## Abstract

Women with false‐positive results are commonly referred back to routine screening. Questions remain regarding their long‐term outcome of breast cancer. We assessed the risk of screen‐detected breast cancer in women with false‐positive results. We conducted a joint analysis using individual level data from the population‐based screening programs in Copenhagen and Funen in Denmark, Norway, and Spain. Overall, 150,383 screened women from Denmark (1991–2008), 612,138 from Norway (1996–2010), and 1,172,572 from Spain (1990–2006) were included. Poisson regression was used to estimate the relative risk (RR) of screen‐detected cancer for women with false‐positive versus negative results. We analyzed information from 1,935,093 women 50–69 years who underwent 6,094,515 screening exams. During an average 5.8 years of follow‐up, 230,609 (11.9%) women received a false‐positive result and 27,849 (1.4%) were diagnosed with screen‐detected cancer. The adjusted RR of screen‐detected cancer after a false‐positive result was 2.01 (95% CI: 1.93–2.09). Women who tested false‐positive at first screen had a RR of 1.86 (95% CI: 1.77–1.96), whereas those who tested false‐positive at third screening had a RR of 2.42 (95% CI: 2.21–2.64). The RR of breast cancer at the screening test after the false‐positive result was 3.95 (95% CI: 3.71–4.21), whereas it decreased to 1.25 (95% CI: 1.17–1.34) three or more screens after the false‐positive result. Women with false‐positive results had a twofold risk of screen‐detected breast cancer compared to women with negative tests. The risk remained significantly higher three or more screens after the false‐positive result. The increased risk should be considered when discussing stratified screening strategies.

## Introduction

Abnormal findings on screening mammograms lead to recall for further assessment, which include additional imaging procedures, and if considered necessary fine needle aspiration cytology, core needle biopsy, or surgical biopsy. Women recalled for further assessment without having a breast cancer diagnosed are considered to have had a false‐positive screening result. False‐positive results are a concern of mammographic screening as they might cause distress, anxiety, and other psychological problems to the women 1, 2. It also implies additional hospital visits, and diagnostic tests, as well as additional costs 3.

The rates of false‐positive screening results depend on the screening performance and organization, such as the screening interval, single versus double reading, participation patterns, sensitivity of the radiologists performance, equipment, and characteristics related to the screening population 4, 5, 6, 7. In Europe, the cumulative risk of a false‐positive screening result after ten biennial screens in women aged 50–69 is estimated to be 20% in a pooled analysis 8. The cumulative estimates for the United States are substantially higher, ranging between 42% for women biennially screened for 10 years from age 50, to 61% in women screened annually for 10 years from age 50 9, 10, 11.

Previous studies have reported an increased risk of breast cancer later in life among women with false‐positive screening results 12, 13, 14, 15, 16, even when taking misclassification into consideration, that is, that some positive results were evaluated to be negative while later detected cancer revealed that they were positive at the time of screening 17, 18. The elevated risk may be related to the increased risk of breast cancer among women with benign breast lesion 19, 20, 21, 22.

Population‐based screening programs are usually run under a “one size fits all” model, without any specific guidelines for follow‐up of women with radiological abnormalities or false‐positive results. However, stratified screening strategies have been discussed 23, 24. Women with false‐positive screening results represent a group of women with potential for stratification based on their breast cancer risk.

We performed a joint analysis using individual level data from long‐standing mammography programs in Denmark, Norway, and Spain to assess the risk of screen‐detected breast cancer in women with false‐positive screening results as compared with women with negative screening results.

## Methods

### Study population

We performed a joint analysis from three population‐based screening programs in Europe. The study is based on individual level data from the Copenhagen and Funen Mammography Registers in Denmark, the Norwegian Breast Cancer Screening Program, at the Cancer Registry of Norway, and the Spanish Breast Cancer Screening Program. All three screening programs target women aged 50–69, perform biennial screening, and are run according to the European Guidelines for Quality Assurance in Breast Cancer Screening 25. The programs have been described in detail elsewhere 7, 26, 27, 28, 29. Information about the screening examinations of 150,383 screened women in Denmark, (1991–2008), 612,138 in Norway (1996–2010), and 1,172,572 in Spain (1990–2006) comprised the data for the study. The women contributed a total of 6,094,515 screening exams. Data on screening results in Denmark were retrieved from the Copenhagen and Funen Mammography Registers. Cancer data were supplied by the Danish Cancer Registry and the Danish Breast Cancer Cooperative Group. Permission for data analysis was granted by the Danish Data Inspection Agency. Screening data in Norway was provided by the Cancer Registry of Norway. Data collection followed the regulations of the institution and no ethical committee approval was necessary since all data used were anonymized. Data from the Spanish Breast Cancer Screening Program was obtained from the screening units databases and received the approval of the Review Boards of the institutions that provided data.

#### Mammography screening in Denmark

Population‐based screening mammography in Denmark started in 1991 in the municipality of Copenhagen, and in 1993 in the county of Funen, and it is state‐funded. The women are invited to a screening examination by personal letter. From the onset of the screening program to 2001, two‐view mammography was performed at prevalent screens. Subsequent screens included one view for women classified with fatty breast tissue at prior screen and two views for women with mixed/dense breast tissue 30. Two‐view mammography was gradually implemented from 2001, and in 2004 all women were covered. Two trained breast radiologist interpret the screening mammograms. At subsequent screens, prior mammograms are retrieved for comparison. Women are classified as negative or positive after mammographic interpretation. Women classified as positive are recalled for additional assessment, mostly using a triple test consisting of clinical examination, mammography, and ultrasound and, if needed, needle biopsy. If consensus still could not be reached, the women are referred for surgical biopsy 7. Women with no breast cancer diagnosed after further assessment are referred back to routine screening, whereas women diagnosed with breast cancer are referred for treatment.

#### Mammography screening in Norway

The Norwegian Breast Cancer Screening Program (NBCSP) started as a pilot in four counties in 1996 and became nationwide, covering all 19 counties in 2005 28. The Cancer Registry of Norway is responsible for the administration, surveillance and quality assurance of the program. Own cost for women is 35–40 Euro, which covers screening and eventually further assessments and treatment. Women are invited by personal letter to a two‐view bilateral mammography. The screening mammograms are independently read by two trained breast radiologists. Prior mammograms are retrieved for comparison at subsequent screens. A score, 1–5, is given for each breast by both radiologists, where 1 indicates a negative screening exam and 5 indicates high susceptibility of a malignancy. All women with a score of 2 or higher by one or both readers are discussed at a consensus meeting where the final decision on whether to recall the woman or not is taken. Further assessments include clinical examination, additional imaging, ultrasound, and invasive procedures (fine‐needle aspiration cytology, core needle biopsy and/or surgical biopsy) if needed. Recall examinations take place at 16 breast clinics at University or County Hospitals, 1–2 weeks after the screening examination. If no malignancy is stated, the women are referred back to screening. All women diagnosed with breast cancer are referred to treatment.

#### Mammography screening in Spain

Population‐based screening in Spain started in 1990 in one region and became nationwide in 2006. Breast cancer screening in Spain is state‐founded. The program is organized into 17 screening settings, responsible for the local application of screening in their area 27. This study included data from eight of the 17 screening settings. Women are invited to participate in the screening program by written letter to a two‐view mammography. Screening mammograms are interpreted by trained breast radiologists. Four of the eight screening settings performed double reading, whereas the remaining four performed single reading. Approximately 36% of the screening exams were read by one radiologist only 31.Prior mammograms are retrieved for comparison at subsequent screens. Screening mammograms are classified according to the BI‐RADS (Breast Imaging‐Reporting and Data System, American College of Radiology, US) scale or equivalent 26. Women with abnormal mammographic findings are recalled for further assessments, including additional imaging, ultrasound, and/or invasive procedures. If no malignancy is stated, women are referred back to screening, whereas women diagnosed with breast cancer are referred for treatment.

### Statistical Analysis

A false‐positive screening result was defined as a recall for further assessment where no breast cancer was confirmed, regardless of the procedures performed (additional imaging and/or invasive procedure with benign outcome). A screen‐detected cancer was defined as breast cancer (ductal carcinoma in situ (DCIS) or invasive cancer) diagnosed as a result of further assessment due to abnormal findings on the screening mammogram interpretation. The rate of screen‐detected cancer was calculated as number of screen‐detected breast cancers divided by the number of screening tests. The false‐positive rate was calculated as number of women with a false‐positive screening result divided by the number of screening tests.

Poisson regression was used to estimate the relative risk (RR) and the 95% confidence interval (CI) of screen‐detected breast cancer for women with a false‐positive screening result compared with women with negative screening tests. The number of incident screen‐detected breast cancer cases was analyzed as a log‐linear function of exposure time (t), exposure status (s), age at screen (a), calendar year at screen (y), and country (c). The model was expressed as ln(*λ*_say_) = *α *+ ln(t_say_) + *β*_s_s + *β*_a_a + *β*_y_y + *δ*_c_; where *α* is the intercept, ln(t_say_) is the time offset of the Poisson regression model given by the log of exposure time, *β* is the slope of the regression line for the covariates in the model, and *δ* is a country‐specific random effect to account for the correlation among screening tests performed in the same country. Exposure status (s) was divided into negative test or false‐positive screening result. We adjusted for age at screen and calendar year at screen (continuous variables). The country‐specific random effect had three levels (Denmark, Norway, and Spain). The outcomes of interest were all screen‐detected breast cancers (DCIS and invasive cancer), invasive breast cancer, and DCIS.

Person‐years at risk were calculated from the date of first screen. Women contributed person‐years at risk to the screened‐negative group as long as the screening tests were negative only. Women contributed person‐years at risk to the false‐positive group from the date of the first false‐positive result. The women were censored at date of breast cancer diagnosis, or last screening date in the file, whichever came first. Women with a breast cancer diagnosis at prevalent screen, and women with one screen only did not contribute person‐years at risk as they were censored at the date of first screen. We performed four separate regression models for each outcome of interest, where false‐positive results were categorized as (1) presence of a false‐positive result (negative test or false‐positive screening result), (2) age at false‐positive result (negative test, false‐positive at 50–54, false‐positive at 55–59, false‐positive at 60–64, or false‐positive at 65–69), (3) screening number at false‐positive result (negative test, false‐positive at first screen, false‐positive at second screen, or false‐positive at third screen or more), and (4) number of screens since false‐positive result (negative test, one screen since false‐positive result, two screens since false‐positive result, or three screens or more since false‐positive result). All tests were two‐sided with a 5% significance level. Statistical analyses were conducted in R 3.0.2 (R Foundation for Statistical Computing, Austria) and SAS 9.2 (SAS Institute Inc, Cary, NC).

To test whether irregular screening participation (women with at least one missing exam in the biennial mammogram schedule) may have an effect on the estimates, we performed a sensitivity analysis where we restricted the calculations to women with regular screening participations only.

## Results

We analyzed data from 1,935,093 women aged 50–69 years. Overall, 230,609 women (11.9%) had a false‐positive screening result during the study period and 27,849 women (1.4%) had a screen‐detected breast cancer, of which 22,694 (81.5%) were invasive cancers, and 4,243 (15.2%) DCIS. The crude rate of screen‐detected cancers was 6.7 per 1000 screening exams in Denmark, 5.4 in Norway, and 3.7 in Spain (Table 1). The crude rate of false‐positive screening results was 16.8 per 1000 screening exams in Denmark, 27.8 in Norway and 46.9 in Spain.

**Table 1:** Characteristics of the study population by country. Women screened age 50–69 years

	Denmark	Norway	Spain
Women, No	150,383	612,138	1,172,572
Screens, No	524,538	2,061,269	3,508,708
Screen‐positive results, No (‰)	12,342 (23.5‰)	68,519 (33.2‰)	177,597 (50.6‰)
Screen‐detected Cancers, No (‰)	3,525 (6.7‰)	11,189 (5.4‰)	13,135 (3.7‰)
Invasive	3,066 (5.8‰)	9,214 (4.5‰)	10,414 (3.0‰)
DCIS	459 (0.9‰)	1,975 (1.0‰)	1,809 (0.5‰)
Unknown	0 (0.0‰)	0 (0.0‰)	912 (0.3‰)
False‐positive results, No (‰)	8,817 (16.8‰)	57,330 (27.8‰)	164,462 (46.9‰)
Screen number, No (%)			
First	150,383 (28.7)	612,138 (29.7)	1,172,572 (33.4)
Second	111,988 (21.3)	488,106 (23.7)	914,354 (26.1)
Third	88,038 (16.8)	382,128 (18.5)	656,710 (18.7)
Fourth	67,976 (13.0)	277,482 (13.5)	408,915 (11.7)
Fifth	50,237 (9.6)	165,358 (8.0)	204,515 (5.8)
Sixth or more	55,916 (10.7)	136,057 (6.6)	151,642 (4.3)
Age at screen, 5y, No (%)			
50–54	154,063 (29.4)	621,060 (30.1)	923,244 (26.3)
55–59	146,440 (27.9)	581,349 (28.2)	1,063,204 (30.3)
60–64	122,390 (23.3)	495,777 (24.1)	1,074,873 (30.6)
65–69	101,645 (19.4)	363,083 (17.6)	447,387 (12.8)
Year of screen, No (%)			
1990–1994	67,586 (12.9)	0 (0.0)	235,690 (6.7)
1995–1999	169,714 (32.4)	240,816 (11.7)	922,495 (26.3)
2000–2004	169,584 (32.3)	686,317 (33.3)	1,597,907 (45.5)
2005–2010	117,654 (22.4)	1,134,136 (55.0)	752,616 (21.4)

The average follow‐up was 5.8 years (5.8 years for women with negative test and 5.6 years for women with false‐positive screening results). Women with negative tests contributed 8,240,964 women‐years at risk, whereas women with false‐positive screening tests contributed 844,323 women‐years at risk. The number of screen‐detected cancers in women with previous negative screening tests was 14,242, giving an absolute breast cancer rate of 1.73/1000 women‐years at risk, whereas the number of breast cancers among women with previous false‐positive screening results was 2691, giving an absolute rate of 3.19/1000 women‐years at risk. The crude rate ratio of breast cancer in women with false‐positive screening results compared with women with negative screening tests was 2.16 in Denmark, 2.00 in Norway, and 2.04 in Spain (Table 2).

**Table 2:** Crude rates of screen detected cancer (DCIS and invasive cancer), and rate ratios for women with false‐positive screening results compared with women with negative results

	Women‐years	No of cases^a^	Crude Rate (‰)	Crude Rate ratio
Denmark				
Negative test	749 785	2 033	2.71	Ref.
False‐positive result	39 847	233	5.85	2.16
Norway				
Negative test	2 840 381	6 388	2.25	Ref.
False‐positive result	208 474	939	4.50	2.00
Spain				
Negative test	4 650 798	5 821	1.25	Ref.
False‐positive result	596 002	1 519	2.55	2.04

a Number of breast cancer cases does not equal the number of screen detected cases in the screening program as breast cancer cases diagnosed at prevalent screen and women with only one screen were censored at date of first screen.

**Figure 1:**
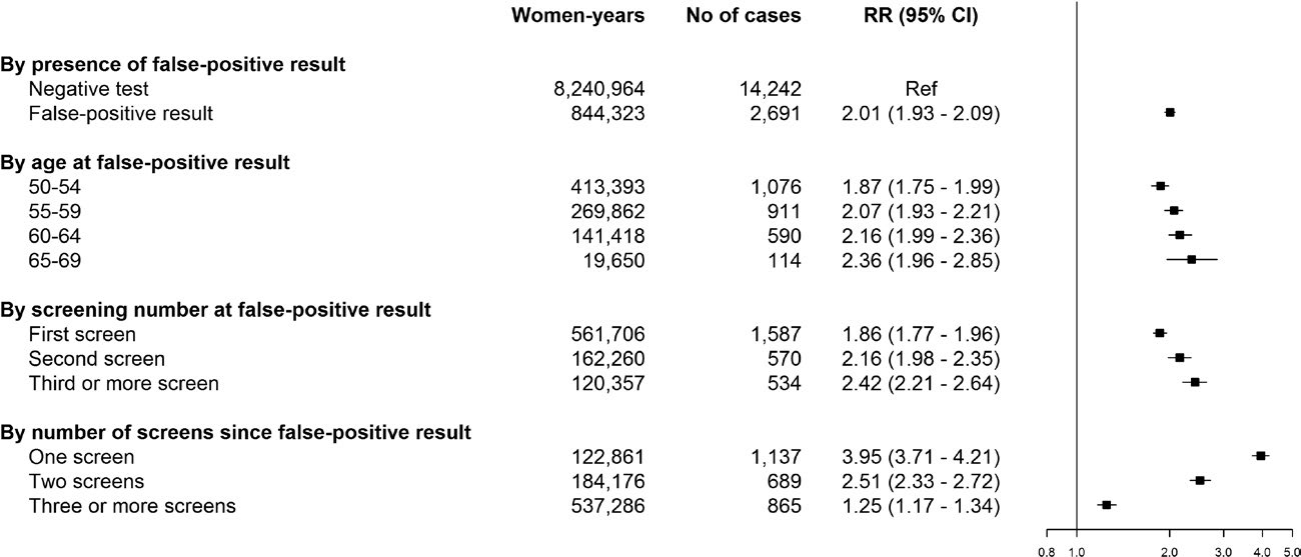
Adjusted relative risk of screen‐detected breast cancer (DCIS and invasive cancer) for women with false‐positive screening results versus women with negative screening tests. CI, confidence interval; RR, relative risk; DCIS, ductal carcinoma in situ. The figure is based on four separate regression models for presence of a false‐positive result, age at false‐positive result, screening number at false‐positive result, and number of screens since false‐positive result. All models adjusted for age at screen, calendar year at screen, country (random effect), and time (offset).

The adjusted relative risk of breast cancer in women with false‐positive results was higher than for women with negative tests (RR = 2.01; 95% CI: 1.93–2.09, Figure 1). The relative risk of breast cancer for women with false‐positive screening results increased by age at false‐positive result; 1.87 (95% CI: 1.75–1.99) for the youngest age group and 2.36 (95% CI: 1.96–2.85) for the oldest. Women with a false‐positive result at first screen had a lower risk of breast cancer compared with women with a false‐positive result at later screens (first screen: RR = 1.86; 95% CI: 1.77–1.96; second screen: RR = 2.16; 95% CI: 1.98–2.35; third screen or more: RR = 2.42; 95% CI: 2.21–2.64). The risk decreased with the number of screening examinations since the false‐positive result. The highest risk was observed in the screening examination following a false‐positive result (RR = 3.95; 95% CI: 3.71–4.21), whereas the RR was 2.51 (95% CI: 2.33–2.72) after two screens, and it was 1.25 (95% CI: 1.17–1.34) three or more screens after the false‐positive result.

**Figure 2:**
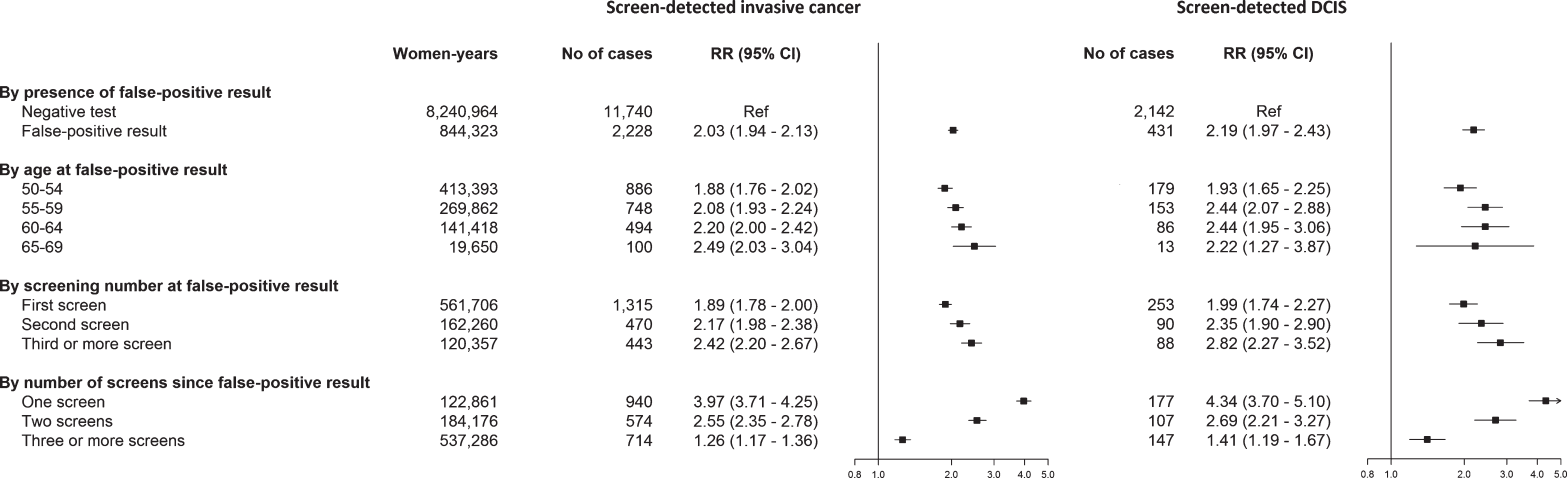
Adjusted relative risk of screen‐detected breast cancer by histologic subtype (DCIS or invasive cancer) for women with false‐positive screening results versus women with negative screening tests. CI, confidence interval; RR, relative risk; DCIS, ductal carcinoma in situ. The figure is based on four separate regression models for each histologic subtype. Separate models for presence of a false‐positive result, age at false‐positive result, screening number at false‐positive result, and number of screens since false‐positive result. All models adjusted for age at screen, calendar year at screen, country (random effect), and time (offset).

The analysis for screen‐detected invasive breast cancer alone, showed similar results as for all malignancies (DCIS and invasive). The relative risk of invasive breast cancer in women with false‐positive results was 2.03 (95% CI: 1.94–2.13) compared with women with negative tests (Figure 2). Results of the analysis for screen‐detected DCIS were also similar to the overall analysis and analyses for invasive breast cancer, except for the risk of screen‐detected DCIS associated with age at the false‐positive screening result, which did not increase with increasing age (Figure 2). The relative risk of screen‐detected DCIS was 1.93 (95% CI: 1.65–2.25) for women aged 50–54 at false‐positive screening result, 2.44 (95% CI: 2.07–2.88) for women aged 55–59, 2.44 (95% CI: 1.95–3.06) for women aged 60–64, and 2.22 (95% CI: 1.27–3.87) for women aged 65–69.

The results excluding women with at least one irregular screening participation (*n* = 174,134) showed a similarly increased risk of screen‐detected breast cancer in women with false‐positive results (RR = 2.00; 95% CI: 1.91–2.09) compared to women with false‐positive results in the full database analyses.

## Discussion

In this joint analysis of data from three population‐based screening programs in Europe, we found that women with false‐positive screening results had a twofold higher risk of screen‐detected breast cancer than women with negative tests. The risk increased with increasing age at false‐positive result, screening number at false‐positive result, and decreased with the number of screens since false‐positive result.

The three screening programs analyzed follow the European Guidelines for Quality Assurance in Breast Cancer Screening and share similar management policies 25. The study population differs in each country with respect to the number of women analyzed, but the follow‐up of screened women is similar, using individual level data with information on all screening tests performed in the program for each woman. The analyses are based on long‐term follow‐up and the data analyzed included information from the start of each screening program (Denmark, 1991–2008; with 6.9 years of average follow‐up; Norway, 1996–2010, 6.0 years of average follow‐up; and Spain, 1990–2006, 5.5 years of average follow‐up).

Despite the similarities in management and data structure across the screening programs, the crude detection rates, and crude false‐positive rates varied across countries. International variation in the detection rates and rates of false‐positive results have been reported previously and were as expected 32, 33, 34, 35, 36. Despite the variation found in the crude rates, the rate ratios of screen‐detected cancer in women with false‐positive results compared to women with negative tests were similar across the three countries. In a sensitivity analysis aimed at testing the consistency of the country effect, we found that the RR in women with false‐positive results was 1.84 in the baseline unadjusted model, 2.03 in the baseline country‐adjusted model (including country as a fixed effect), and as shown in the results section, it was 2.01 in the fully adjusted model (including country as a random effect). The model testing for consistency showed that the country variable had little effect on the overall estimate.

Several studies have reported an excess risk of breast cancer in women with false‐positive screening results 12, 13, 14, 15, 16, 17. One of the first studies on the topic was carried out in the Netherlands in 1988 by Peeters et al. 17. In a mean follow‐up of 5 years, they found a relative risk of breast cancer of 2.72 for women with a false‐positive screening result. In the context of population‐based screening programs in Europe, McCann et al. found a higher risk of breast cancer detection at the second screen in women with a false‐positive result at first screen (OR = 2.15) which is consistent with the results of this study 15. However, it should be noticed that the study was carried out in the United Kingdom, where the screening interval is 3 years compared to the 2‐year interval in most population‐based screening programs in Europe. In contrast to our study, Groenendijk et al. did not find any significant differences in risk of breast cancer in women with false‐positive results compared with women with negative tests 14. However, the study was limited by a small sample size. In addition, the screening program in the Netherlands have reported a low recall rate and high‐positive predictive value, which might be of influence. A study from the Copenhagen program in Denmark by von Euler‐Chelpin et al. found a 67% increased risk of breast cancer diagnosis in women with false‐positive results 16. Castells et al. also found an increased risk of screen‐detected breast cancer in women with false‐positive results (OR = 1.81) 13. However, the data used in the Danish and Spanish studies are partially included in this study and thus, no further comparison is done to avoid bias. An increased risk of breast cancer has also been reported in the context of opportunistic screening in the United States, where Barlow et al. found an increased risk of breast cancer (OR = 1.69) within 1 year after the last screening test in women with a false‐positive result 12.

A false‐positive result at first screen gave a lower relative risk than a false‐positive result at later screens. This might be explained on one hand, by the higher recall rate at initial screen which may be associated with radiological findings lest attributable to a breast cancer risk. On the other hand, it may be explained by the availability of earlier mammograms for comparison at subsequent screens, which is a common practice in the three screening programs, and leads to identification of more true positives at subsequent screens. Women with false‐positive results at first screen might thus be a less selected population than those with false‐positive results at subsequent screens.

Previous studies have shown that in population‐based screening programs women with false‐positive results may be less likely to re‐attend subsequent screening invitations 37, 38, which could underestimate the risk. However, in the analyses including women with regular screening participations only, the risk estimate was similar to the estimate for the full study population, indicating that the effect of irregular screening participation is negligible in our study.

The highest risk of screen‐detected cancer was found in the screening test immediately following a false‐positive result, and decreased gradually with the number of screens since false‐positive result. However, the risk remained statistically significant three or more screens after the false‐positive result. The decreasing risk might indicate some misclassification of breast malignancies at baseline assessment. On the other hand, the long‐term risk supports the idea of a biological susceptibility for developing breast cancer in women with mammographic findings leading to a recall for further assessments were malignancy is finally ruled out. Previous studies have discussed the role of misclassification in women with false‐positive screening results 17, 18. von Euler‐Chelpin et al. found that 24% of women who later developed breast cancer after a false‐positive result were misclassified at baseline assessment 18. Peeters et al. argued that in 75% of the breast cancers in women with a false‐positive result, the malignancy was present at the baseline referral 17. The four‐fold increased risk found at the first screen following the false‐positive result favors the hypothesis of misclassification. Misclassification should be assessed related to laterality and location of the suspicious finding leading to a false‐positive result and the breast cancer. Unfortunately, data on laterality and location was not available as part of the information gathered for this study. However, it is remarkable that the increased risk remained significantly higher three or more screens after the false‐positive result, which favors the hypothesis of biological susceptibility. This is consistent with the excess breast cancer risk found in women with benign breast lesions 19, 20, 21, 22. Women with false‐positive results at screening represent a selected population of women with radiological abnormalities at baseline assessment.

This is, to our knowledge, the first study to analyze the risk of screen‐detected DCIS and invasive breast cancer in women with previous false‐positive results. No substantial differences were found in the risk for DCIS and invasive cancers compared to the overall analyses, which may indicate that false‐positive results are not related to a specific histological subtype of breast cancer. In addition, we analyzed the breast cancer risk in women with false‐positive results using a joint data base with individual level data of three population‐based screening programs in Europe. The availability of complete data from the screening programs enabled us to map exposure and outcome on an individual level, with no loss to follow‐up. However, this study also had some limitations. The analyses were restricted to screen‐detected breast cancer cases only. We had no information on interval cancers and breast cancers detected outside the screening program. False‐positive results are likely to increase not only the risk of screen detected cancers but also the risk of clinically diagnosed breast cancers, which would have been desirable to analyze 16. Restricting the analysis to screen‐detected cancers only, implies that the follow‐up time is shorter for women in the oldest age group and for those women with a first false‐positive result in later screens compared with women with a false‐positive result in earlier screens. Also, we lacked information on which exams lead to a biopsy recommendation, which would have added interesting information on the risk associated with false‐positives including an invasive procedure with benign outcome. In addition, the histological subtype for DCIS and invasive cancers was missing for approximately 2.3% breast malignancies. This lack of information affects only the specific analysis for invasive and DCIS cancers. However, the results obtained were very similar for DCIS and invasive cancers, so this lack of information was not deemed important.

The negative effects of false‐positive results have been widely noted and include the anxiety of being recalled for further assessment 1, which may discourage women from participating in subsequent screening invitations 38, 39. Based on the findings in this study it might be advisable to actively encourage women with false‐positive results for regular screening participation, as their potential benefit from screening is higher than in women with negative tests. To achieve this goal, women with false‐positive results could be provided with specific‐targeted information about their increased risk and the benefits of regular screening participation when the woman is informed about the negative results from the additional tests to rule out malignancy.

In conclusion, we found that women with false‐positive results had a twofold risk of having a later screen‐detected breast cancer. The risk remained significantly increased three or more screens after the false‐positive result. The increased risk should be considered when exploring the possibilities of stratified screening to optimize effectiveness and minimize the harms of organized mammographic screening.

## Conflicts of interest

The authors have declared no conflicts of interest.
